# Dental Medicine

**Published:** 1892-02

**Authors:** 


### Bibliographical.

DENTAL MEDICINE.—A Manual of Dental Materia Medica and Therapeutics. By Ferdinand J. S. Gorgas, A. M., M. D., D. D. S., Professor of the Principles of Dental Science, Dental Surgery, etc., in the University of Maryland, etc. Fourth edition. Revised and enlarged. Philadelphia, P. Blakiston, Son & Co., 1891. Cincinnati, Robt. Clarke & Co., pp. 524. Price, cloth, $3.50.

This is probably recognized by the dental profession as the most complete and valuable work on dental medicine. Considerable matter has been added in this edition pertaining especially to the diagnosis of the affections of the mouth and the differential remedial agents. A new chapter has been added on the use of antiseptics which includes the sterilization of instruments. A number of new and valuable formulæ have been added, and the Index to "Dental Diseases and Remedies," has been materially increased. The subject of inflammation occupies some twenty-six pages and it is presented in a most acceptable manner, the whole subject being pretty well gone over.

The author's classification and remarks on anæsthesia are especially well prepared. The entire work gives evidence of experience as well as careful and discriminating judgment on the part of the author.—*Cincinnati Med. Journal.*

